# Developing an ethical framework for the recruitment of people who inject drugs experiencing incarceration in HIV prevention research: a qualitative study

**DOI:** 10.1186/s12954-024-01138-z

**Published:** 2024-12-20

**Authors:** Matthew Murphy, Nyx Gomes, Kimberly Kane, Josiah D. Rich, Lloyd Goldsamt, Jasjit S. Ahluwalia, Kate M. Guthrie, Susan E. Ramsey, Sara Vargas

**Affiliations:** 1 Lifespan, Providence, RI USA; 2 Alpert Medical School of Brown University, Providence, RI USA; 3 Brown University School of Public Health, Providence, RI USA; 4 Rhode Island Department of Corrections, Cranston, RI USA; 5 New York University, New York, NY USA; 6 DGIM-Research, Rhode Island Hospital, 111 Plain Street, First Floor, Providence, RI 02903 USA

**Keywords:** HIV, PrEP, Incarceration, Research ethics, Qualitative research, Substance use

## Abstract

**Background:**

HIV disproportionately impacts people who experience incarceration. Incarceration represents an opportunity to engage in HIV prevention care for individuals who often experience a number of barriers accessing health services in the community. The development of evidence-based practices promoting pre-exposure prophylaxis for HIV prevention (PrEP) is crucial for ending the HIV epidemic within this highly marginalized population. However, PrEP research within carceral facilities has been limited and is hampered in part by the lack of ethical guidance on conducting HIV prevention research in this unique setting where incarcerated individuals are categorized as a vulnerable population requiring specific protections. This lack of knowledge is particularly striking when considering the lack of input from incarcerated individuals themselves on the responsible conduct of research, which is critical to understanding ways to ensure participant autonomy while avoiding coercive practices in research activities.

**Methods:**

In order to gain a better understanding of ethical approaches to the conduct of HIV prevention research among incarcerated individuals, we conducted qualitative interviews with 21 incarcerated men who reported injecting drugs and met clinical criteria for PrEP use. The interview topics included HIV knowledge, PrEP knowledge, stigma, and perceptions related to ethical research practices.

**Results:**

Themes identified included how forced abstinence during incarceration can negatively affect research participation, the importance of participant comfort as it relates to ensuring autonomy in decision making, a desire for person centred approaches in research activities, study staff characteristics impacting participant experience, and perceptions of carceral staff as members of research teams.

**Conclusions:**

The results of this study indicate that conducting research focused on improving PrEP use in a carceral environment has support among those experiencing incarceration. However, researchers should place the participant experience at the center of research protocol development.

**Supplementary Information:**

The online version contains supplementary material available at 10.1186/s12954-024-01138-z.

## Introduction

People who inject drugs are at high risk for HIV acquisition and represent a substantial percentage of new HIV infections in the United States (US) [1]. Incarcerated individuals are disproportionately impacted by substance use disorders including injection drug use (IDU) and HIV [5, 6]. Recent and ongoing outbreaks of HIV among people who inject drugs underscore the importance of developing new interventions to prevent HIV transmission among this vulnerable group [2, 3]. The criminal legal system provides an important, yet underutilized, public health opportunity to link individuals at high risk for HIV acquisition to HIV prevention services [4]. Importantly, individuals are at extremely high risk of HIV acquisition immediately after release from incarceration [7, 8].

One promising HIV prevention approach is pre-exposure prophylaxis (PrEP), a medication that is effective in preventing HIV among people who inject drugs [9, 10]. Despite its efficacy, and the unique public health opportunity that the criminal legal system provides to engage people who inject drugs for PrEP care [11], little is understood about how to improve PrEP initiation in the carceral setting, as well as adherence and linkage to care upon community re-entry.

Research to develop evidence-based approaches to increase PrEP use and reduce HIV transmission in the vulnerable period after release from incarceration would benefit from input by people who inject drugs experiencing incarceration [12]. At the same time, incarcerated populations are uniquely vulnerable and require special protections during the conduct of research [13]. Perhaps the most notable concern is incarcerated individuals’ reduced autonomy, as well as their vulnerability to undue influence and coercion to participate in research activities [14]. People who inject drugs also experience unique vulnerabilities and ethical considerations when participating in HIV prevention research [15], requiring particularly rigorous ethical protections for this target population.

Despite this marked vulnerability, and the availability of community-engaged frameworks for the protection of vulnerable human subjects in research [32], there has been little research that has incorporated perspectives from incarcerated individuals exploring potential mechanisms to ethically enroll individuals in research who are detained within the criminal legal system [16]. Additionally the limited research available on ethical practices in research study design within the criminal legal system shows high variability in research practices [17]. Prior research has also underscored the concern for incarcerated individual’s decision-making capacity, as well as the potential impact of coercive influences [18]. Of particular concern is the general lack of input from incarcerated populations themselves on what they view as the most ethical and responsible practices for conducting research in this uniquely complex environment [19].

Given the need for more evidence-based approaches to address the disproportionate impact of the HIV epidemic on this uniquely vulnerable population, the need for additional research is clear. At the same time, the special ethical considerations for conducting research among those experiencing incarceration [14], the vulnerability of people who inject drugs including concerns for safety and undue inducement [20], and the difficulties in collecting effective data, require the development and clear characterization of ethical approaches to study design in HIV prevention research with input from individuals experiencing incarceration. This qualitative study addresses the lack of community engagement on ethical approaches to conducting HIV prevention research including PrEP care in the carceral setting by collecting formative data with the goal of developing a community-engaged ethical framework for the conduct of research promoting PrEP use among populations experiencing incarceration as they return to the community.

## Methods

Semi-structured qualitative interviews were conducted among men who reported a history of injecting drugs (N = 21) and were currently experiencing incarceration. Interviews averaged approximately 45 min in length (range 20–85 min) (note: one participant declined to continue with the interview after 4 min due to disinterest in the interview after the first few questions and thus was not included in the average). Interviews were conducted with participants experiencing incarceration at the Rhode Island Department of Corrections (RIDOC). RIDOC is a unified carceral system where individuals in the State of Rhode Island awaiting trial, as well as those sentenced to a period of confinement, are detained. It is located on one campus and managed under a singular, publicly funded security and medical administration, and is one of six state carceral systems that lacks local or county facilities with distinct administrative entities. Also notable is that RIDOC is among the first states to describe its implementation of PrEP care [21, 22] as well as support ongoing PrEP related research [21–23]. This study, all of its materials, and methods were approved by the Lifespan Institutional Review Board and the RIDOC Medical Research Advisory Group.

To be eligible to participate, an individual must have been incarcerated at the time of the interview, at least 18 years of age, have a history of IDU, be clinically indicated for PrEP according to CDC guidelines, identify as a cisgender man, and be able to understand and speak English and to provide written and verbal informed consent. Recruitment of people who inject drugs experiencing incarceration occurred at the men’s intake facility managed by RIDOC. Every individual upon commitment to RIDOC undergoes a nurse-led intake evaluation to assess health needs, including management of substance use disorders and HIV risk. The rigorous intake process includes questions about a history of IDU, severe substance use disorders, and other health processes that are more prevalent among people who inject drugs (e.g., Hepatitis C infection). Based on this intake screening, as well as encounters with other clinical staff such as physicians and substance use treatment providers, individuals who reported a history of IDU and are deemed at increased risk of HIV acquisition are referred to a PrEP provider for further clinical evaluation. Individuals identified as part of these routine clinical processes who may meet the inclusion criteria were referred to study staff within the carceral facility where they were offered the opportunity to enroll in the study. Of note, the study PI (Dr. Murphy) was also the principal PrEP prescriber at RIDOC during the course of this study and was not directly involved with study referral, recruitment, consenting, or other study activities to avoid placing any undue influence on potential study participants.

Individuals who were offered the opportunity to enroll were called to the clinical area during standard healthcare operating hours and were led to a private clinical room with a research staff member. Research staff explained the purpose of the study and explained that participation was voluntary before asking if an individual would like to complete a study screener to determine eligibility (verbal consent to be screened). Participants who met inclusion criteria and wished to participate were then further briefed on the study objectives, provided with a comprehensive review of the consent form, and had a chance to ask any questions about the study prior to signing the consent form. This process involved several reminders that the study was voluntary and that participants were free to leave at any time. All participants signed consent forms, as well as verbally affirmed that they had been properly informed about the study when signing the consent form.

Interviews aimed to collect formative data by exploring four broad areas related to the conduct of HIV prevention research in the carceral settings that were also informed by the key ethical principles identified in the landmark Belmont report: (1) perspectives on participating in HIV prevention research during a period of incarceration, including potential benefits, risks, and drawbacks; (2) aspects of perceived vulnerability during a period of incarceration, including access to health services and substance use disorder treatment; (3) interactions with clinical and security staff during the course of routine clinical care and implications for research participation; and, (4) preferences for mechanisms, timing, and amount of reimbursement (see Appendix 1 for example questions).

Interviews were audio-recorded by research staff, transcribed by a HIPAA-certified transcription company unaffiliated with the carceral setting, and reviewed for accuracy by research staff. The 21 transcripts and 21 standardized debriefing forms containing interviewer notes were coded and analyzed using the framework method [24] with a deductive codebook informed by the goals of the project. Interview data were independently coded by two coders, with overall coding concordance greater than 85%. In addition, the team reviewed coding applications during team meetings for consensus and an agreed-upon set of codes for each transcript was applied. Data were summarized by code into a framework matrix, a widely used approach in formative multi-disciplinary qualitative health research, [24] utilizing NVivo software [25]. The identification of themes was guided by the ethical research principals described in the Belmont Report [26]. Themes were established by review and further reduction of the data within the matrix by members of the research team. Of note, those members include author MM, study PI, internal medicine physician and PrEP clinical provider within RIDOC who designed the study, was responsible for oversight of the research team, and reviewed deidentified qualitative data; author NG, research assistant, undergraduate degree in psychology, who was involved with data collection, reduction and analysis; all author authors were responsible for mentoring and training the PI and the team in design and conduct of the study, and reviewed all drafts of this manuscript..

## Results

### Participants largely favor conducting public health-related research in the carceral setting

Most participants (19 of 21) were supportive of research activities being conducted in the carceral setting. One participant exemplified this frequently expressed sentiment saying:

“What yous are doin’, [members of the research team], is very good. I mean, it’s gonna help a lot of people in the long run once it’s up and runnin’ and people really understand that yous are tryin’ to help, and it’s not about bullshit.” (participant [P]1: African American, gay, early 50s, IDU greater than one year ago)

Of the participants who were supportive of research, a few (4 of 21) explicitly stated that research provided a positive contribution to an otherwise challenging experience, noting that it could, in and of itself, provide an opportunity for HIV prevention education and access to important information on PrEP. After being asked about the provision of research materials like those offered as part of this study, one participant talked at length about both liking the research and feeling like it was an opportunity to access educational resources saying:

“I like the whole idea of it [PrEP pamphlet] and getting people to participate in the study and feel comfortable doing it. It’s big. It’s definitely good to get it out there and known. I know this study is more about how to approach people to do the study rather than the prescription itself, but I had no idea about the PrEP. It’s something I’ll definitely let people know about.” (P19: White, heterosexual, late 20s, IDU in last three months)

Only one participant felt generally negatively about participating in research activities while incarcerated, largely due to a distrust of medical systems and a perceived misallocation of resources in drug development.

### Forced abstinence during incarceration and substance use disorder management affect potential research participation

About half of participants (11 of 21) reported that individuals experiencing incarceration who are withdrawing from substance use are unlikely to want to participate in research while symptomatic. Many of these participants believed that the decision to participate in research may be influenced by the withdrawal symptoms resulting from the involuntary cessation of substance use, or forced abstinence, while incarcerated, a frequently occurring phenomenon. Some participants shared past experiences and the experiences of those around them, characterizing withdrawal as extremely unpleasant, describing symptoms such as fatigue, nausea, personality shifts, anxiety, and general discomfort. When describing the experience of withdrawal and how that could affect participation in research activities, one participant stated:

“Especially if they’re sick, and they’re not gonna wanna talk about it. They’re not gonna—not until they feel like they can be more where they’re not sick, to be honest with you. When you’re sick, you don’t wanna do anything. You don’t care about anything. You can’t concentrate.” (P13: white, heterosexual, late 30s, IDU within the last three months)

Withdrawal symptoms and substance use disorder treatment were recurring topics throughout the course of study interviews. Some participants reported that their opioid agonist therapy was affecting their conversation during the interview by making them anxious and uneasy. Others reported becoming extremely tired and distracted during the interview, leading to an early conclusion to their research participation. This was the case with P20:

“Right now, I’m sorry, I can’t really think because I took my methadone.” (P20: Hispanic, heterosexual, mid-20s, IDU within the last three months)

Several participants (5 of 21) thought researchers should avoid recruiting individuals who were withdrawing by not attempting to recruit individuals who had been incarcerated for less than a week. For example:

“I’d give them a week. Check when their intake was, so on and so forth. I first got here, and you knew in an instant I was a drug user, and so on and so forth. I’d give them a week to at least be not sick anymore and start to have their mind back ‘cause if I was sick right now, I probably would’ve denied the interview.” (P5: white; bisexual; late 30s; IDU within the last 3 months),

This sentiment was echoed by another participant:

“I think that they have to wait like 7 to 10 days before they are able to take suboxone or methadone, before they can get somethin’ that will be a comfort med to ‘em where they’ll feel better. You don’t want nobody near that’s gonna be dope sick.” (P12: white; heterosexual; late 40s; IDU within the last 12 months)

It should be noted that one participant dissented from this opinion, believing that most people withdrawing would like to be interviewed and that they should be considered for potential participation in research.

Strategies to address participants experiencing withdrawal symptoms were proposed. Participants suggested making the amount of time research activities would take clear from the outset and highlighting the voluntary nature of study participation. Another suggestion was to include carceral healthcare staff in discussions related to identifying potential research participants to help researchers avoid those who were experiencing substantial withdrawal symptoms. Additionally, some study participants suggested that, if research staff do come across someone who they think is withdrawing, researchers should refer that person to healthcare staff prior to potential enrollment in a research study. Further supporting participant statements, study staff noted that some individuals displayed behaviors consistent with withdrawal symptoms during interviews, at times impacting the openness to participating in interviews, as well as the information shared during the interview process.

### The importance of participant comfort as it relates to autonomy

Within the carceral setting, participants highlighted specific challenges and considerations related to participant autonomy that would need to be addressed in order to ensure the ethical conduct of research. The carceral setting was characterized as an environment that leads to inherent restrictions related to individual autonomy, causing potential study participants to be constantly on guard. Due to this pressure, participants suggested elements of study design, particularly for interviews, that would enable researchers to provide comfort to participants, thereby strengthening the autonomy of potential participants to make decisions related to research participation. These included providing snacks and drinks to participants, conducting research activities in a one-on-one format, and clearly communicating the potential uses of a study, any benefits that participants might expect or not, important health information relevant to research activities, and, in the case of HIV prevention/PrEP research, the relevancy of PrEP to the participant’s life. To the contrary, some participants noted that they did not perceive any pressure to participate in research and thus no specific measures were required to address that potential concern. Further, some participants noted that certain pressures, such as social and legal, can be unavoidable while incarcerated and that researchers should tactfully address those pressures when conducting research related activities. For example, one participant explained why one-on-one research interactions were important to him:

“One-on-ones, yeah, generally ‘cause you’re gonna get skewed answers if you don’t otherwise… There would definitely have to be privacy…” (P18: white, early 40s, gay, IDU within the past three months),

The idea of keeping interviews short, even if that meant conducting multiple interviews over time, with or without compensation, was popular among participants. Additionally, several participants expressed an interest in post research activity check-ins as a way to demonstrate continued interest in participant well-being, as well as to provide updates on the progress of the study. Participant 12 said:

“I think there should be some kind of counseling or weekly or biweekly check-in to sit down and talk and see how everything’s going.” (P12)

Participants also noted that researchers should take into account other scheduling elements for research activities to avoid impacting important routines in the carceral setting. As an example, several participants noted that research should avoid interrupting meal time, referred to as “chow”. Participant 5 said:

“Try to do it between—I don’t know if you could find out when feedings are. Try to stay away from those periods… Try to see if that block [housing unit] has eaten yet or going to chow.” (P5)

Some participants suggested increasing comfort measures during research activities to alleviate any pressure a participant might perceive and help build rapport, for example:

“Hey, I don’t know if they’ll let you do that, but if I have soda here, a can of soda or water and I have like chips or candy, something like that, we are restricted in jail. We can’t just go get food. That’s why I’m saying money. If you come in and you’re broke, like me, I’m hungry at night. If I come in here and I’m eating, I’m gonna be comfortable with you. I’m gonna develop a bond right there. This kid, this guy [interviewer] or whatever is bringing me food and candy just to talk to him. Even though I’m not gonna get paid, it helps.” (P8: white, heterosexual, early 40s, IDU within the past three months)

### Person-centered approaches to study design and research activities

A few participants (3 of 21) mentioned that they felt like the limited pay they receive for work tasks, as well as carceral facilities’ access to their information without their input, are indicative of the larger carceral system as exploitative. This quote expresses how one felt dehumanized and exploited:

“A lot of us just feel like another number where the state doesn’t actually care about fixing people or whatever. They just want another number to make them money.” (P6: white, heterosexual, early 30 s, IDU greater than 1 year ago).

Study participants expressed that researchers can address these concerns by incorporating a person-centered approach and avoiding treating participants as numbers or a resource to be exploited. Participants noted that treating people in a dehumanizing way would discourage volunteers from participating in research and negatively impact the researcher-participant relationship. When asked about what sorts of things would make him not want to participate in research, one participant responded by expressing that he would like to be viewed as an individual by saying:

“I guess being another statistic. That’s the only thing I could see anyone getting turned off by.” (P6).

Participants also noted that researchers should be sensitive to an individual’s emotional state to avoid research encounters leading to subsequent negative or hostile interactions between participants and others while incarcerated. This includes focusing on rapport-building and patient-centered interview approaches used by study staff. When discussing how emotions and research intersect, participants revealed a myriad of potential feelings around, and motivations for, participating in research. Participants often express that they have little to do while incarcerated and that research can be a break from the monotony of being incarcerated. The sentiments of ‘it’s something to do’ and ‘it gets me out of my cell’ were common motivations that were often mentioned when initially hearing about the interview or during the consent process. Additionally, a few participants (4 of 21) expressed that they wanted to feel like their participation in a study will benefit other people. Participants largely wanted, and liked, seeing some sort of potential positive effect of their research participation, describing their motivation to participate in research as altruistic. When discussing why a person who is incarcerated might want to participate in research, one participant expressed an altruistic motivation:

“The shitty things that I’ve done all my life if anything good can come out of it, then sure.” (P7: Hispanic, bisexual, mid-40 s, IDU within the last three months).

A few (4 of 21) participants also expressed the importance of research’s relevance to them and using it as an opportunity to learn more about things that may benefit their health. One participant combined multiple motives when he said:

“I would do it ‘cause it’ll be helping other people and also give me a little bit more knowledge on it and something I’m involved in. I would like to participate.” (P15: Hispanic, heterosexual, late-30 s, IDU within the last three months).

### Import of characteristics of study staff and experiences as research participants

Several participants expressed a mistrust of academic information, or information provided by researchers, and placed more trust in anecdotal personal experiences of known social contacts. One participant, for example, suggested that sharing information on the effectiveness of a medication being studied in the carceral setting, in this instance PrEP, is another way to increase comfort, and build trust with research staff, noting that he would like to see proof of PrEP’s effectiveness, saying:

“I would literally have to see someone that I know taking it, and literally I went and tested it, and fuck some bitch with HIV, or AIDS or whatever, and come back and tell me that they don’t have it, ‘cause seeing is believing most of the time.” (P6)

Several (6 of 21) participants felt that researchers should be relatable or have some sort of personal experience with the research topic or population at hand. When talking about the importance of researchers having lived experience, one participant talked about how learning through lived experiences was more important than knowledge learned more formally:

“Give people real-life experiences, not textbook shit. I keep goin’ back to the textbook, textbook ‘cause it’s always been a problem with me—people—the textbooks—they just read out of books. I could read anything out of a fuckin’ book. Until I experience it, I don’t know what the fuck’s goin’ on. I don’t know what they’re talkin’ about. It doesn’t matter. To me, it just doesn’t matter.” (P16: white, heterosexual, early 50s, IDU more than 1 year ago),

Specific to HIV prevention, other suggestions included participants interacting with research staff who have personal experience in taking PrEP and thus acting as proof that the medication is effective, or having research staff who have a personal tie to HIV.

The perceived similarity between study staff and potential research subjects in the carceral setting was noted as positive by several participants. Study staff who seem less academic and more “street-smart” seemed to be perceived as more able to build rapport and ensure fluid communication. One participant suggested that researchers find commonalities between themselves and their participants to help with rapport-building during study sessions. Another suggested that an interviewer who is older than the participants may be beneficial to building rapport, potentially a function of the lived experience participants felt was particularly beneficial. Of note, some participants expressed that they did not know what they would look for in an ideal study staff member because they had little experience participating in research.

Participants also expressed that the demeanor of research staff would affect a participant and their willingness to participate in a study. Kind, respectful, and positive researchers can attract participants where more negative interactional behaviors would dissuade individuals from participating in research, as exemplified by this quote:

“Say, somebody else that came up here and was like, ‘Oh, hour-long interview? Fuck that. I’m going back downstairs,’ which did cross my mind, but it was the vibe. You seem like a cool person.” (P4: white, bisexual, late 20 s, IDU within the last year).

Qualities such as empathy and being a good listener were highlighted as traits that research staff should have. Several participants expressed that they wanted the freedom to talk in interviews, though it is noted that this should be within reason and gentle redirections were appreciated and were understandable given the more specific goals of research interactions. Participants reported appreciating being treated as individuals and noted that research staff should be aware of an individual’s emotional state during the course of research activities and be “open” so that they could be comfortable, e.g.:

“Somebody who’s not like, I don’t know how to—being like sarcastic in a way. Somebody who’s open to being able to talk with you and you feel comfortable talking with, that you’re not feeling like they’re pressuring you to answer the questions and stuff.” (P13).

Other positive attributes of research staff that were noted by participants included being ready for anything and fearless given their acknowledgement of the unique challenges of the carceral environment. Participants valued research staff authenticity, describing qualities such as honesty and genuineness as being important. Further, participants noted a preference for knowledgeable research staff who were independent from the carceral setting. One participant noted that some study participants may have a gender preference when it comes to those conducting some research activities, including in-depth interviews.

### Participants were divided on carceral healthcare staff involvement in research activities

There was a range of perspectives regarding carceral healthcare staff involvement with research. Some participants (5 of 21) were amenable to DOC healthcare staff participating in research activities, as exemplified in this quote:

“They should be involved. They should be involved. They should know about this.” (P11: mixed-race, heterosexual, early 50 s, IDU more than 1 year ago).

Some participants saw DOC healthcare staff as being particularly useful in prescribing and distributing PrEP. Others, as noted previously, thought that carceral clinical staff could identify potential candidates for research while helping to avoid potential participants who were withdrawing or have other pressing health needs. This led a few participants (3 of 21) to express more ambivalence on carceral clinical staff involvement and noted that they should only be on a need-to-know basis when it comes to research:

“Oh, for them, I guess, is, what would they need to know? Other than just like that I want it. Other than that, it’s like, what do they need to know? Unless they need to pass off information. It really, I guess, depends on … what they need to know, on like a need-to-know basis.” (P6).

Of the several participants who felt it was appropriate to involve carceral healthcare staff in research, a recurring sentiment was that their participation should not impact, or distract from, their main clinical responsibilities. Indeed, some (7 of 21) of the participants expressing this belief felt strongly that healthcare staff should not be involved in research activities, particularly because of other health needs that needed to be addressed by clinical staff. Other participants went further, expressing the view that they were an extension of the security apparatus and thus should not participate in health-related research activities. One participant was adamantly opposed to carceral clinical staff being involved in research, noting:

“No. You need more staff. If there’s gonna be research from [carceral] staff, they need to bring in professionals from [hospital 1] and things like that. Not staff from up here ‘cause these staff that work here as medical professionals, they clean, they be around the COs [correctional officers] too much, and they start to feel as though they got the authority.” (P14: black, heterosexual, early 30s, IDU less than 6 months ago)

### Participant perceptions regarding carceral security staff involvement during research activities

Carceral security staff, namely correctional officers, referred to as “COs,” were brought up in interviews either by participants that mentioned them spontaneously or in interviews where officers may have inserted themselves in the interview environment or been in the vicinity. Routine functions of the carceral facility where this study took place, which fell to COs to enforce, also led to interruptions in interviews that occasionally caused interviews to be divided into more than one session. For instance, during one, there was an interruption by a CO when the participant was required to pick up an order from the commissary.

Participants noted concerns that a CO’s presence or involvement could be disruptive to research activities in a number of ways, leading to an individual altering their decision to participate in research activities or altering the information that they would share during the course of an interview or other research encounter. Participants expressed concerns about CO’s overhearing confidential research conversations and were concerned by the privacy of the room in which interviews took place. The space where research activities take place and the privacy afforded by that space were noted as key priorities by participants, as illustrated by this quote:

“The privacy of this room is a little concerning. I wish it was a closed—I wish there weren’t guards circling. I know the most you can get is a closed door. You can’t close that curtain. I know. I constantly feel as though they’re eavesdropping on us ‘cause that’s their job.” (P18)

## Discussion

**Fig. 1:**
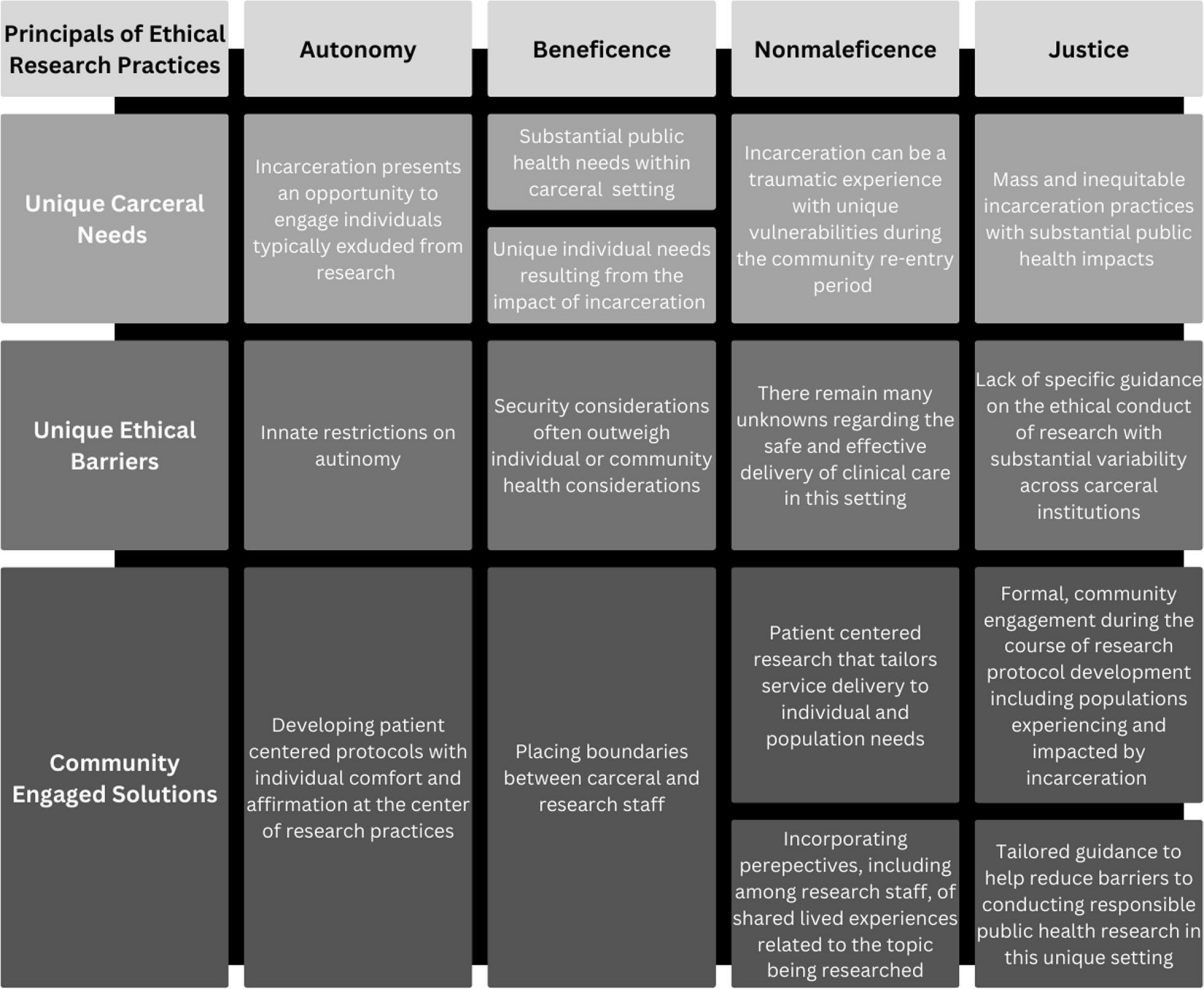
Ethical principals in carceral research

Results from this study contribute critical considerations for the responsible conduct of research in carceral settings, as the voices of those experiencing incarceration are frequently not included in the peer-reviewed literature. They have also been used to create an original framework to help interpret results as well as chart future directions in ethical research practice in this setting (see Fig. 1). It is important to note that, overall, participants were very receptive to, and encouraged the availability of, HIV prevention research during incarceration. Having community support for this area of research is critical to both its ethical foundation and its successful completion. Additionally, the study’s results help to address concerns that carceral administrators might have regarding the interest of incarcerated populations in having access to HIV prevention research, as well as PrEP clinical care more broadly. The results also align with core ethical principles described in the landmark Belmont report regarding the importance of avoiding undue influence and coercive activities, although this study’s qualitative data provide greater detail and context into ways that research might incorporate ethical, and avoid unethical practices from the perspective of potential research participants. This is particularly true for individuals with a history of substance use disorders, a particularly vulnerable group during the period of incarceration, given periods of forced abstinence and varying degrees of access to substance use treatment services including opioid replacement therapy in carceral settings.

 While there were several notable findings from this study, perhaps the most notable, particularly when thinking about the development of ethical research enrollment practices, were the perspectives shared about the impact of withdrawal symptoms resulting from forced abstinence on participation in research, as well as how it might influence research-related activities in this setting. On the one hand, several individuals noted that potential participants should not be approached until they have had several days to address the symptoms of forced withdrawal. On the other hand, particularly in the jail setting where there is high turnover and relatively short incarceration stays [27], (Centers for Disease Control and Prevention (CDC) 1998), excluding this population from research would mean potentially not tailoring health services to their needs and missing the potential public health impact of interventions geared towards this population. In fact, the number of individuals impacted by being jailed far exceeds those who are incarcerated in the US’s prison facilities, which have substantial public health impacts broadly but also specific impacts on HIV transmission [7, 8] and substance use outcomes [5, 6]. The experiences captured in this study indicate that special considerations would be needed if research aims to recruit and study this particularly vulnerable population. In many ways, RIDOC is unique as being one of the first states to implement opioid replacement therapy throughout the state’s carceral system [28], so the experience of incarcerated populations in other states may differ based on the availability of substance use treatment services.

There were also fairly consistent perspectives shared on the importance of researchers working in this space to make potential research participants comfortable and the research experience person-centered. Specific examples of potential approaches were also suggested, including providing snacks and drinks as well as ensuring a welcoming environment. Participants consistently noted the importance of the empathetic treatment of incarcerated individuals by research staff, which has been previously highlighted in the qualitative research literature as an important facilitator of meaningful interview interactions [29]. This underscores the importance of approaches to staffing research teams that include individuals with a history of lived experience of incarceration and/or substance use, although there are frequently limitations placed on individuals with a history of incarceration from accessing carceral facilities. Yet, empathetic treatment is critical to ensuring a positive experience and reducing some of the innate pressures to participate in activities that many individuals experience or perceive while incarcerated that diminish individual autonomy. This can be particularly true as it relates to the decision to participate in research while incarcerated. The lived experience of research staff and their ability to build rapport while empathetically communicating with study participants was also a recurring theme underscored as important by study participants. These are important considerations, particularly as researchers consider the selection and training of staff to work within carceral settings and with individuals impacted by the experience of incarceration. At the same time, not all suggestions from participants would be feasible in this setting or would likely contradict superseding policies particularly related to safety and security within the facility where the study was conducted. Researchers should more systematically engage individuals experiencing incarceration prior to the initiation of research conducted in the carceral space to provide more tailored and nuanced community-engaged perspectives that can then be incorporated into conversations with carceral administrators and clinical staff to determine what approaches are the most ethical and feasible to incorporate. Standardized national guidelines on specific elements of study protocols may be difficult to implement in the diverse array of local, country, state and federal carceral facilities.

Additionally, plans for the conduct of research activities should including specific considerations for the space used within a carceral facility. Often, this will require some sort of negotiation and approval from carceral administrators that aligns with security protocols. However, researchers should aim to be able to use spaces within carceral environments where potential participants will feel confident that the information they disclose will remain confidential and where any potential conflicts with carceral staff can be minimized, if not avoided all together. The consideration of space is also directly related to developing study protocols that focus on research participants’ emotional status surrounding research participation to ensure that participant autonomy is reinforced in this space and that the security of research staff is assured, key to ensuring a positive research interaction. This can include how study information is communicated to potential research participants, the structure and frequency of research encounters, and other details to ensuring a more affirming space that is conducive to participant-centered encounters. To that end, researchers should consider how study results are communicated to participants post-participation and consider the inclusion of supportive or social encounters outside of research encounters to demonstrate the commitment to individual and population well-being in this setting.

There was a spectrum of opinions about the involvement of carceral health care staff during the conduct of research, although some important roles, such as identifying individuals who might be good candidates for research, were noted. Participants underscored the importance of research not distracting staff from their primary health care function and only being involved in aspects of research that were absolutely necessary for the safe and effective conduct of research. Others felt that, by involving health care staff in research activities, carceral staff would benefit from some of the inherent educational and monitoring activities that are part of research. Public health researchers are often tasked with working with carceral health care administrators to develop and receive approval for research protocols, thus thoughtful considerations of health care staff involvement should be a part of those conversations. At the same time, researchers may be limited in their ability to dictate the involvement, or lack thereof, of carceral health care staff based on carceral systems’ competing priorities or staffing policies. There may be benefits to strategically involving outside health care staff for research activities, when possible, given some of the concerns expressed by participants. Regardless, researchers and carceral administrators should work to ensure that they avoid both the real and perceived exploitation of protected health care information from populations with reduced autonomy or control over the health services they receive while incarcerated. As such, access to carceral health data should be specific to the study at hand and researchers should limit the use of potentially protected or identifiable information. This is a particularly important point given the unique HIPAA status of health information collected during the course of incarceration which is, unlike other healthcare entities, frequently generated from nonvoluntary encounters and may be disclosed with fewer protections [30]. Researchers therefore need to ensure they only utilize identifiable health information that is voluntarily offered as part of study activities for which individuals have provided consent. There needs to be careful balancing of the need to protect individual anonymity with any population benefits from study activities, which can also include aggregated or deidentified data, although these were not themes explored in depth during the course of this study. Future research can help to characterize community perspectives on the use of different approaches to accessing and analyzing carceral healthcare data for research purposes.

Study participants shared perspectives related to the involvement or presence of security staff that have been described in ethics related research in carceral settings previously [31]. However, here again, the data provided by participants and even the experience of study staff, underscore the very real challenge posed by conducting research in this setting. Security priorities in this setting generally take precedence over other considerations. Yet, privacy considerations, particularly related to the presence and role of security staff in enforcing carceral security policies, was noted as a concern when considering research participation. The presence and involvement of security staff during the conduct of research activities was likely to impact the ability and ease of sharing potentially sensitive information. Security policies and facility schedules will need to be followed by research staff while being incorporated into study protocols. At the same time, efforts should be made to negotiate with carceral administrators to use space that ensures privacy while adhering to security protocols and ensuring the safety of research staff. Additionally, researchers should be aware of, and aim to develop protocols around, facility schedules so that research participants do not miss crucial, scheduled activities such as meals, legal and personal visitation times.

While the themes that emerged during the course of this study are formative, they align with community-engaged research ethics frameworks that note the need to contemplate the potential risks to participants during the conduct of research [32]. Namely, there are unique potentials for research processes to introduce risks to an individual’s well-being and the potential for research activities to negatively impact an individual’s autonomy within this setting. The ethical principles outlined in the landmark Belmont Report help to frame the nuanced community-engaged results which can inform the future ethical conduct of research within carceral settings (Fig. 1) [33].

## Limitations

This study does have some limitations. As with all ethical research conducted, individuals who declined to participate in this study may have substantially different views on the responsible conduct of research in this setting. Of note, we recruited cisgender men, the most prevalent population incarcerated in the United States. Other groups, particularly women and gender minority populations, may have different perceptions related to the ethical conduct of research in carceral settings. Additionally, some of the unique characteristics of the study setting may make the findings difficult to generalize to other carceral settings. As an example, all of the individuals who participated in this study were recruited from the “awaiting trial or jail” facility. There may be important differences shared by individuals who are detained in prison facilities. Additionally, RIDOC is only one of six unified state carceral systems in the US, potentially leading to distinct administrative and health service delivery activities for potential research subjects experiencing incarceration. Although some participants offered spontaneous, positive feedback about the involvement of the study PI in clinical activities, the dual role of clinician-researcher, which has previously been discussed in carceral settings [34], could be further explored in greater detail. The clinical research environment within RIDOC is unique as the study PI is not formally carceral staff but an external contracted physician who provides PrEP clinical care within RIDOC facilities, further underscoring the importance of community and stakeholder engaged ethical study design in this unique setting. Future research should also explore community perspectives on specific study designs which could include differential intervention or control groups as well as study status assignation given incarcerated individual’s reduced autonomy, as well as access to health care services. Finally, given the unique role of compensation for participation in research activities in this setting, a detailed exploration of community informed compensation practices is also of critical importance and should be the focus of subsequent ethics-oriented studies.

## Conclusion

There is a critical need for HIV prevention research within the carceral setting and there is support among those experiencing incarceration for conducting this research in this setting. Researchers should aim to hire and train staff so that potential research participants are comfortable and are able to “connect” with researchers during the course of research activities. While developing study protocols and requesting approval from carceral administrators, researchers should take into account security policies and facility schedules to ensure participant comfort, privacy, and ability to participate in other scheduled activities. Thoughtful consideration of the role of carceral health care staff should be part of these discussions with carceral administrators, as well as ways to ensure that security policies are followed while allowing for private and safe research activities with participants. While future research is needed on specific details and types of research activities, this study provides key insights into how HIV prevention research can be ethically conducted in carceral settings, particularly those that are detaining individuals for brief periods of incarceration.

## Supplementary Information

Additional file 1.

## Data Availability

No datasets were generated or analysed during the current study.
