# Supplementary figures and images for: Developing an ethical framework for the recruitment of people who inject drugs experiencing incarceration in HIV prevention research: a qualitative study
Source: Harm Reduct J. 2024 Dec 20;21:223. doi: 10.1186/s12954-024-01138-z (PMC11660855; doi:10.1186/s12954-024-01138-z)

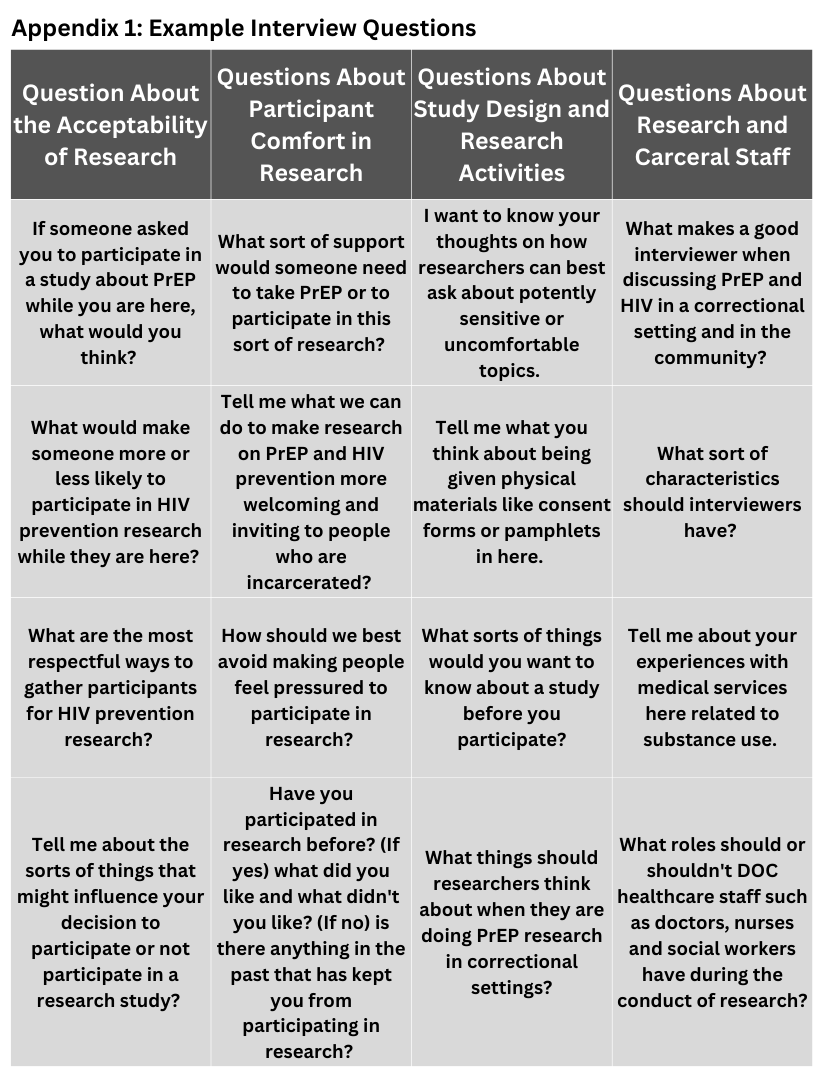

Supplement: Supplementary file 1 — Additional file 1. [file 12954_2024_1138_MOESM1_ESM.png]
